# Hierarchical porosity *via* layer-tunnel conversion of macroporous δ-MnO_2_ nanosheet assemblies†

**DOI:** 10.1039/c9ra08432k

**Published:** 2020-01-08

**Authors:** Peter C. Metz, Alec C. Ladonis, Peng Gao, Trevyn Hey, Scott T. Misture

**Affiliations:** NYS College of Ceramics, Alfred University, Kazuo Inamori School of Engineering Alfred NY 14802 USA misture@alfred.edu

## Abstract

This work reports the layer-tunnel conversion of porous dehydrated synthetic alkali-free δ-MnO_2_ analogs prepared by exfoliation, flocculation, and heat treatment of nanosheets derived from highly crystalline potassium birnessite. High surface area porous solids result, with specific surface areas of 90–130 m^2^ g^−1^ and isotherms characteristic of both micro and macropores. The microstructures of the re-assembled floccules are reminiscent of crumpled paper where single and re-stacked nanosheets form the walls of interconnected macropores. The atomic and local structures of the floccules heat treated from 60–400 °C are tracked by Raman spectroscopy and synchrotron X-ray total scattering measurements. During heating, the nanosheets comprising the pore walls condense to form tunnel-structured fragments beginning at temperatures below 100 °C, while the microstructure with high surface area remains intact. The flocc microstructure remains largely unchanged in samples heated up to 400 °C while an increasing fraction of the sample is transformed, at least locally, to possess 1D tunnels characteristic of α-MnO_2_. Cyclic voltammetry in Na_2_SO_4_ aqueous electrolyte reflects the nanoscale structural evolution, where intercalative pseudocapacitance diminishes with the degree of transformation. Collectively, these results demonstrate that it is feasible to tailor the materials for applications incorporating nanoporous solids and nanofluidics, and specifically imply strategies to maintain a kinetically accessible interlayer contribute to Na intercalative pseudocapacitance.

## Abstract 2

This work reports the layer-tunnel conversion of porous dehydrated synthetic alkali-free δ-MnO_2_ analogs prepared by exfoliation, flocculation, and heat treatment of nanosheets derived from highly crystalline potassium birnessite.

## Graphical abstract

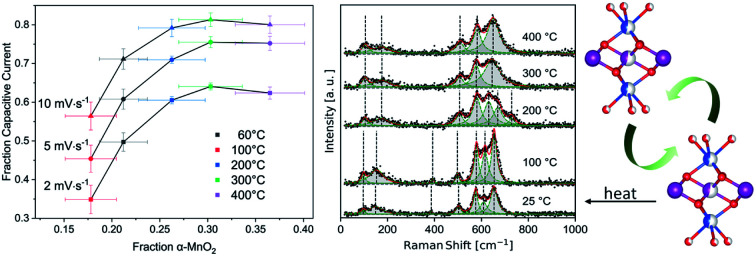

## 1 Introduction

Transition metal oxide nanosheets provide a promising approach to fabricating nano- and meso-structured materials through topochemical modification,^1^ exfoliation,^2^ reassembly,^3^ and subsequent processing steps. The exfoliation-assembly route can yield ordered films,^4^ as well as numerous mesostructured ensembles with morphologies including nano-flowers, nanorods, crumpled newspaper, and *etc.*^5^ In the particular case of MnO_2_, these mesoporous ensembles are stable in temperature as high as 4–500 °C, after which thermodynamic competition of the surface and bulk yields fascinating nanoscale coral-like structures. Further, manganese dioxides are known to exhibit controllable porosity at the nanometer length scale *via* control over phase formation amongst the tunnel-structured MnO_2_ polytypes.^6,7^ Consequently, it is conceivable to design δ-MnO_2_ nanosheet ensembles with hierarchical porosity across length scales spanning several orders of magnitude.

The chemical and structural flexibility of manganates is leveraged in nature and in the laboratory. Various MnO_2_ polytypes appear in biological^8^ and geochemical^9^ processes, and play a critical role in environmental accumulation of valuable or toxic cationic species.^10^ In the laboratory, extensive research has been committed to understanding the electrochemical properties of manganates, both in regard to their energy storage capabilities,^11,12^ and to their catalytic activity.^13–15^ Electrochemical studies highlight ion transport mechanisms in the MnO_2_ polymorphs, where intercalation between δ-MnO_2_ layers and into the 2 × 2 tunnels of α-MnO_2_ is feasible, but smaller tunnels are inaccessible to alkali ions.^16^ Further work has explored both the α and δ polymorphs and even conversions between the δ and α forms.^17–19^ More recently, many catalysis studies comparing the layer and tunnel variants have been reported, with the specific reaction chemistry determining activity.^19–24^

The crucial difficulty in establishing rigorous mechanistic links of the behavior of manganates lies in interpreting the interrelation between the manganese electronic configuration and the extensive polytypism displayed in this phase space, a challenge exacerbated by the nanoscale nature of the material. MnO_2_ polytypes exist in a structural continuum delineated by the extent of edge- and corner-sharing octahedral MnO_6_ units.^25^ In parlance of the spectroscopic community,^26–29^ manganates may largely be classified by the integer numbers (*m*, *n*) of edge-shared MnO_6_ units forming a tunnel structure, denoted T_*m*,*n*_. These include T_1,1_ β-MnO_2_ (pyrolusite), T_2,2_ α-MnO_2_ (cryptomelane), T_3,3_ τ-MnO_2_ (todorokite), and *etc.*^9^ Other important polytypes include lamellar birnessite-like minerals with T_1,∞_ (*e.g.* infinite δ-MnO_2_ planes), and spinels (λ-MnO_2_) which may be viewed as phyllomanganates with ordered interlayer and layer defect structure. Because of this structural proximity, interconversion between these structures is common.^30–34^

**Fig. 1:**
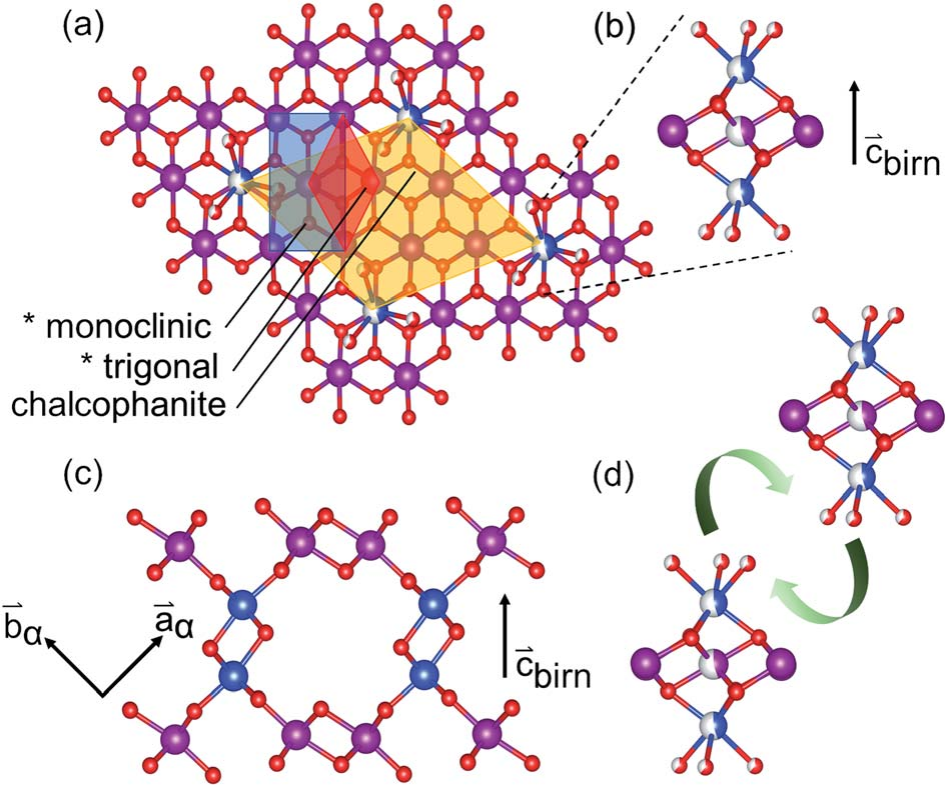
Relationship of the defective birnessite motif (*) in the monoclinic and trigonal settings, and in the defect-ordered chalcophanite model (Mn^3+^ in half-filled blue) (a). Views showing the Ruetschi-type defect (b) and elimination of defect-coordinating water in the interlayer condensation reaction (d) to form the α-MnO_2_ motif. Half-fill indicates partial occupancy. (c) Interlayer linkages forming the tunnel structure.

The sensitivity of Mn species to environmental factors results in the ready oxidation and reduction of manganates. Coupled with the Jahn–Teller distortion of Mn^3+^O_6_, these changes to the Mn electronic configuration result in strain-driven alterations to the edge-shared MnO_6_ network. The classic exposition of this mechanism was given by Ruetschi^35^ in consideration of the physical and electrochemical properties of γ- and ε-MnO_2_. This Ruetschi defect is comprised of the removal of a Mn^3+^ cation from the lattice, transport to an available surface, and coordination by available water species. In T_*m*,*n*_ structures with abundant interior “surface” available, as in the case of T_1,∞_ birnessite-like minerals, reduced Mn^3+^ species need only migrate ∼2 Å, making the formation kinetically accessible (Fig. 1(b)).

Additionally, the structure and polytypism of MnO_2_ materials is sensitive to the presence of impurities (*e.g.* alkali, alkaline earth, Zn, Cu, Ag, and *etc.*) which play a critical role in selecting the product phase by stabilizing various MnO_2_ frameworks.^36–38^ The formation of Ruetschi defects, which are effectively hydrated interlayer-adsorbed cations, are expected to play a similar role in templating phase transformations.^30,31^

In the present work, we detail the conversion of δ-MnO_2_ nanosheet floccs derived from aqueous colloidal processing of potassium birnessite that is nominally defect-free and of high crystal perfection. Thus, the population of Ruetschi defects is controlled by the aqueous processing conditions and the conversion reaction is not impacted by electrochemical driving forces nor by cations that template tunnel structures. Phase formation of the exfoliated and re-assembled δ-MnO_2_ nanosheet floccs is tracked by *ex situ* laboratory X-ray powder diffraction (PXRD). *Ex situ* Raman spectroscopy (RS), synchrotron powder diffraction (SXRD), and atomic pair distribution function (PDF) data are employed to identify structural changes in representative samples up to 400 °C.

Because the natural mineral analogs generally contain a variety of elemental impurities,^9^ this work will refer to the chemically pure MnO_2_ tunnel structured T_2,2_ phase as α-MnO_2_ to avoid ambiguity. The prototypical structure of the nanosheet is denoted δ-MnO_2_, while specific phyllomanganates are referred to the mineral name (*e.g.* potassium birnessite K_*x*_MnO_2_).

## 2 Experimental

### 2.1 Synthesis

Potassium birnessite was produced by a conventional solid-state method. MnCO_3_ and K_2_CO_3_ were mixed in a 40 : 9 molar ratio by milling in isopropyl alcohol using a McCrone Micronizing Mill (McCrone Group, USA) with alumina media. The milled suspension was subsequently dried on a hot plate for 30 minutes and then calcined in an alumina crucible at 800 °C for 24 hours in air.

H_*x*_MnO_2_ was produced by ion-exchanging the resulting K_*x*_MnO_2_ powder. In a typical batch, 0.5 g of parent birnessite was suspended in 45 mL of 1 N HCl. The suspension was ultrasonicated for 4 hours at room temperature with periodic agitation to reduce sedimentation. The ion-exchanged powders were separated by centrifugation and washed with deionized water three times. This entire procedure was repeated twice for complete K^+^ ion removal. The resulting H_*x*_MnO_2_ was dried and stored for further processing.

Exfoliated nanosheet suspension was obtained by ultrasonicating a suspension of 0.35 g of H_*x*_MnO_2_ dispersed in 32.5 mL of 120 mM tetrabutylammonium hydroxide solution for 4 hours at room temperature, again periodically agitating the suspension to reduce sedimentation. A stable colloidal suspension was obtained by centrifugally separating the unexfoliated material at 10 000 rpm for 10 minutes.

Nanosheet agglomerates were obtained by titrating the nanosheet suspension to pH 2 using 6 N HCl added dropwise, causing the nanosheets to flocculate. The flocculated suspension was equilibrated for 24 hours before centrifugal separation, washing in 2-propanol, and drying at room temperature.

### 2.2 Characterization

Scanning electron microscopy (SEM) of the parent material was carried out with a JEOL 7800F (JEOL, Japan) using an accelerating potential of 20 keV. Low voltage imaging of the floccs was performed at 1–2 keV *via* stage biasing, without coating the samples.

Laboratory powder X-ray diffraction (PXRD) patterns were collected using a Bruker D8 Advance diffractometer with Cu-K_α_ radiation in Bragg–Brentano geometry, a Ni metal filter, and a LYNXEYE XE position-sensitive detector. Thin, unoriented samples were loaded on zero-background off-axis quartz plates and rotated at 30 rpm.

Unpolarized Raman spectra were collected using a WITec Alpha 300RA (WITec GmbH, Germany) equipped with a variable power 488 nm laser and Zeiss EC Epiplan 20× objective. Laser power was fixed at 40 μW to minimize beam damage to the nanosheet ensembles. Spectra were acquired by summing 10 50 μm line scans with 60 s of integration time each. Data were processed with Project FIVE+ software (Version 5.0, Build 5.0.3.43, WITec GmbH, Germany). The Raman scattering profiles were modelled using Lorentzian peak profiles and a constant background term, the parameters of which were optimized using the software package Fityk.^39^ The estimated standard deviations of the refined peak positions were typically 1 cm^−1^.

Isothermal N_2_ adsorption data were measured using a Micromeritics TriStar II Surface Area and Porosity Instrument. The specific surface area (SSA) was calculated using the BET method. Sample degassing is a critical component of the BET measurement and is extremely challenging for the samples of interest which are not thermally stable. Therefore, samples were degassed at room temperature under turbopump vacuum for 3 days before making triplicate measurements. After measuring the SSA for an as-prepared sample, the entire BET tube was heated to the target temperature for layer-to-tunnel conversion and equilibrated for 3 hours, then returned to the BET instrument for a new measurement.

### 2.3 High-energy X-ray scattering

Synchrotron powder X-ray diffraction profiles and atomic PDF data were collected using beamline 11-ID-B of the Advanced Photon Source (Argonne National Laboratory). Data were acquired under ambient conditions from samples sealed in 1.1 mm polyimide capillaries using Si(311) monochromated 58.66 keV (*λ* ≈ 0.2114 Å) radiation in Debye–Scherrer geometry with a PerkinElmer amorphous silicon 2D detector.^40,41^ Medium resolution powder diffraction data were acquired at a detector distance of 950 mm, while data for PDF extraction were acquired at 180 mm.

2D data were corrected for spatial distortions and integrated to obtain 1D powder diffraction profiles using the software package Fit2D,^42^ with CeO_2_ as the calibrant. PDF data were reduced using the software package PDFgetX2,^43^ which enables background subtraction, normalization, and correction of other experimental artifacts.^44,45^

### 2.4 Cyclic voltammetry

#### 2.4.1 Measurement

The electrochemical response of the δ-MnO_2_ nanosheet floccs and the heat-treated products were assessed using cyclic voltammetry. Working electrodes were prepared by mixing 80 wt% active material, 15 wt% acetylene black, and 5 wt% polyvinylidene difluoride in *N*-methyl-2-pyrrolidone solution for 6 hours. The resulting slurry was spread onto a Ni foil current collector with an area of 1 cm^2^ and thickness 16 ± 4 μm. Electrodes consisting of material treated in excess of 100 °C were dried at 100 °C for 2 hours, while active material treated at 60 and 100 °C were dried at 60 °C for 24 hours to evaporate residual solvent and prevent any additional structural changes. All resulting electrodes had typical mass loadings of 0.7–1.0 mg cm^−2^ of active material.

The capacitive performance was investigated using a commercial electrochemical analyzer (CHI 650E, CHI, USA), utilizing a platinum counter electrode and Ag/AgCl reference electrode in a 1 M Na_2_SO_4_ electrolyte. Four electrodes were prepared and tested for each heat treatment step. CV measurements were carried out at 2, 5, 10, 20, 50, and 100 mV s^−1^ scan rates in the voltage window 0–0.8 V. Each electrode was cycled 3 times, using the last cycle for the calculations reported in this study.

#### 2.4.2 Treatment of data

In processing and measurement of these electrodes the uncertainty on the mass of the active MnO_2_ component was substantially larger than the uncertainty on the measured current. We note that (i) the average working electrode had approximate mass and volume of 1 mg and 1 cm^2^ × 16 ± 4 μm respectively, and (ii) we assume that the influence of electrode geometry is negligible compared to the influence of variation in electrode mass. Thus, we assume that the differences in integrated current between replicates are primarily attributable to the variation in electrode mass, while the subtle differences in voltammogram features are the result of any other variability between electrodes.

To clarify the trends in material behavior in the absence of precise mass measurements, we have utilized a dimensionless current *Ĩ* normalized to the integrated current measured for each replicate1
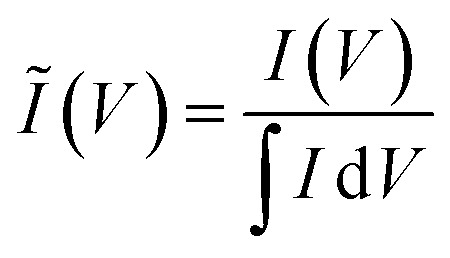
(*I* = measured current, *V* = potential *vs.* Ag/AgCl). The kinetic analysis is carried out using *Ĩ*, yielding an interpretation where the confidence interval reflects the variability of the replicate electrodes prepared for each temperature step. A quasi-specific capacitance *C̃*_m_ [F g^−1^] is obtained by scaling the integrated anodic or cathodic current by the average integrated current2
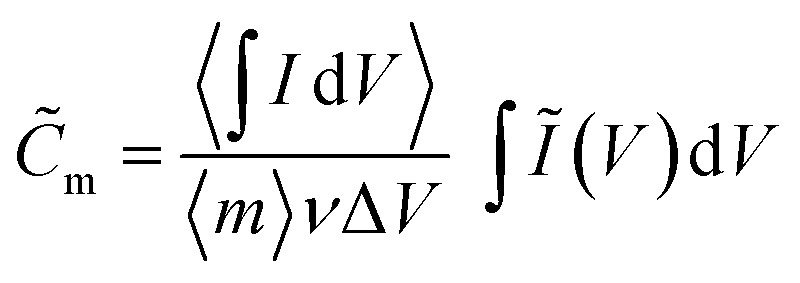
where the average mass of δ-MnO_2_ is 〈*m*〉 = 0.8 mg.

#### 2.4.3 Remarks on theory

Broadly speaking, MnO_2_ exhibits both conventional electrical double layer (EDL) capacitance due to the adsorption of alkali from the supporting electrolyte at the cathode surface, and continuous, reversible faradaic charge storage commonly referred to as pseudocapacitance. As discussed by Brousse^46^ and earlier by Conway,^47^ pseudocapacitive and battery-like behavior are distinguished by their charge and discharge characteristics; namely, that in a capacitor the cycling behavior is reversible, and the current responds linearly with the modulation of applied potential3
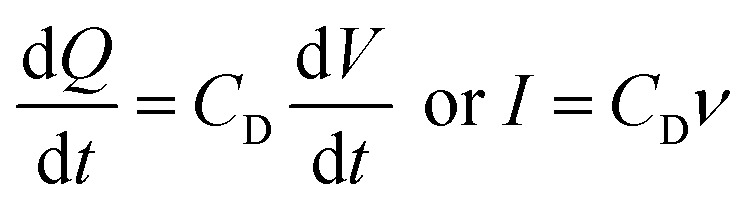
where in cyclic voltammetry *ν* [mV s^−1^] is a constant scan rate, *C*_D_ the gravimetric EDL capacitance, and *I* the current.

Adsorption of the electrochemically reactive species (*e.g.* Na^+^ in this case) may be kinetically limited, either due to experimental artifacts as in the case of depletion of Na^+^ in a dilute supporting electrolyte, or due to sluggish transport in the working electrode. The empirical diffusive current is approximated by solutions to Fick's second law,^48,49^ with a typical functional form4
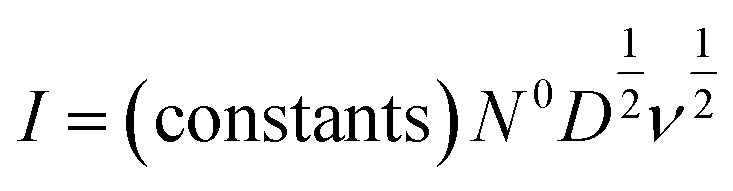
where *N*^0^ is the surface concentration of the diffusant, *D* is a diffusion coefficient, and *ν* is again the potential scan rate.

In real electrodes, the current response in a cyclic voltammetry experiment is generally a combination of capacitive (eqn (3)) and diffusive (eqn (4)) components. The extent to which the charge or discharge current reacts capacitively or diffusively is often assessed by performing a logarithmic regression analysis, assuming the current obeys a power law^49–51^

5 *I* = *av*^*b*^

where *a* and *b* are fitted parameters, and the so-called *b*-value adopts values of 1 for a completely capacitive current and 1/2 for a completely diffusive current. While commonly reported in literature, we note6
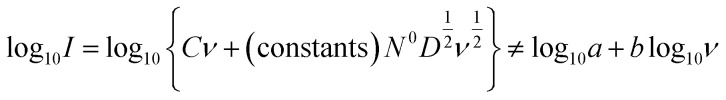
a consequence of which is that the *b*-value retains a functional dependence both on charge state and scan rate. Thus, the power law assumption and the logarithmic regression analysis are only valid over small intervals of scan rate (perhaps a decade or two) and should be compared between studies with care.

Alternatively, the total current can be cast in a linear form by dividing through by *ν* or *ν*^1/2^, yielding7
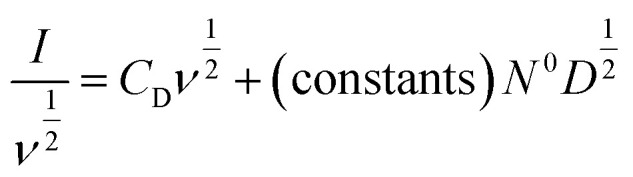
and8
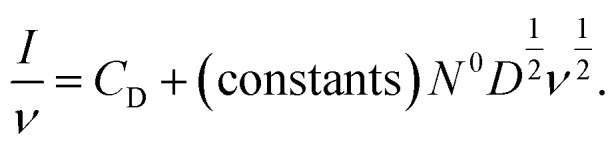
Eqn (7) and (8) are valid for any value of the prefactors, allowing for separation of the capacitive and diffusive currents. The EDL capacitance is nominally constant over the range of sweep rates relevant to capacitive devices. However, the diffusional current should depend upon the dominant transport mechanism, namely intercalative transport in the case of a sufficiently concentrated supporting electrolyte.

## 3 Results and discussion

### 3.1 Sample characteristics

**Fig. 2:**
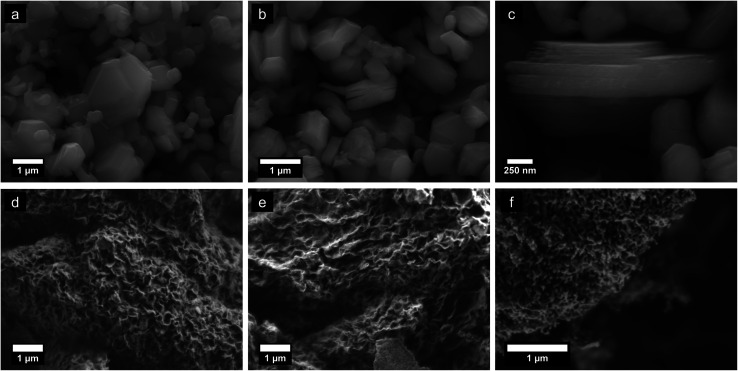
Secondary electron images of K_*x*_MnO_2_ parent compound (a), H_*x*_MnO_2_ ion exchanged powder (b) H_*x*_MnO_2_ ion exchanged powder with magnification highlighting layer delamination (c), and floccs treated to 100, 300, and 600 °C, respectively (d–f).

Morphologies of parent K_*x*_MnO_2_, H_*x*_MnO_2_, and reassembled pH 2 equilibrated floccules are shown in Fig. 2. Potassium birnessite crystallizes as hexagonal platelets, with lateral dimensions of around 1 μm. The ion exchanged phase retains the parent particle morphology but shows swelling along the normal of the plate due to repulsion of the negatively charged δ-MnO_2_ sheets. Flocculated material exhibits an open 3D porous structure with a crumpled-newspaper-like morphology, comprised of reassembled rumpled and bent sheets. The specific surface area of as-prepared samples ranges from 80 to 170 m^2^ g^−1^ with variations deriving from the starting powder particle size. Samples that were milled and then exfoliated reach SSA values of ∼170 m^2^ g^−1^. The adsorption isotherms (ESI†) are characteristic of mesoporous solids.^52^ It is interesting to note that the SSA for the assembled floccules is some 3 to 6 times larger than for at least three variants of nanoscale tunnel-structured MnO_2_,^18,23,24^ though it is possible to make α-MnO_2_ nanowires by hydrothermal growth with SSA approaching 100 m^2^ g^−1^.^20^

Fig. 2(d–f) exhibits the morphology evolution upon heating to 100, 300, and 600 °C. The microstructure remains largely unchanged at temperatures below ∼400 °C. Above this temperature substantial mass diffusion is apparent, leading to formation of a coral-like microstructure with open channels by 600 °C. Direct measurement of the surface area after heat treating shows that effectively no change occurs; for example ESI Fig. S3† shows the adsorption isotherms for an as-prepared sample degassed at room temperature *vs.* one after heat treatment at 300 °C. The SSA for this particular sample is near 80 m^2^ g^−1^ after heating, thus unchanged from the original sample. This result is unsurprising given that the SEM images are essentially indistinguishable for the floccs as shown in Fig. 2(d) and (e).

**Fig. 3:**
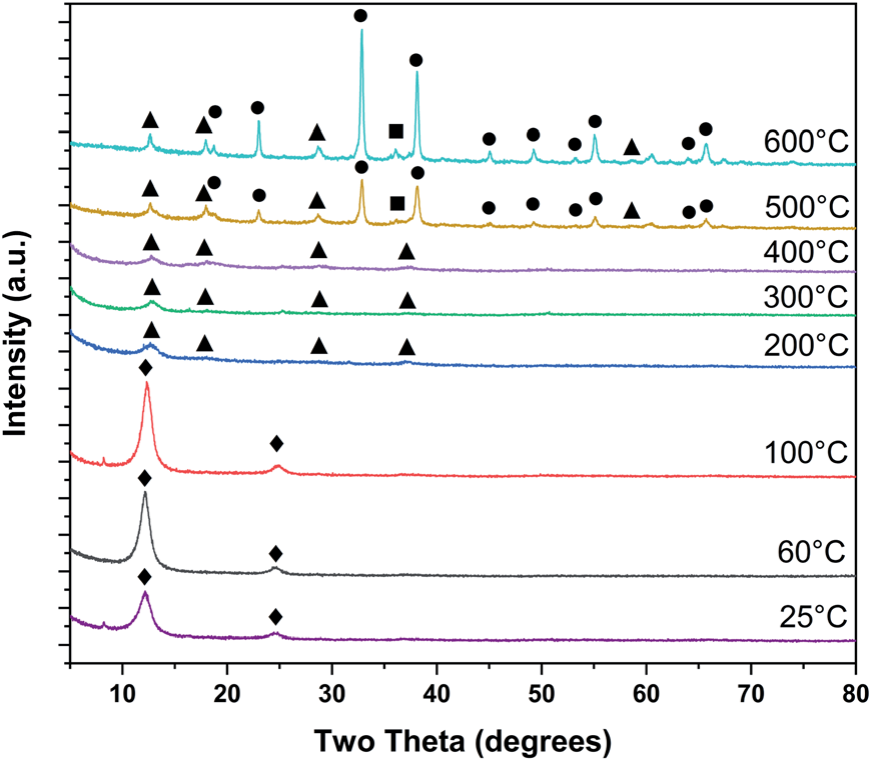
Phase evolution of δ-MnO_2_ nanosheet floccules tracked by *ex situ* laboratory X-ray powder diffraction (♦ δ-MnO_2_, ■ α-Mn_3_O_4_, ● α-Mn_2_O_3_, ▲ α-MnO_2_).

*Ex situ* PXRD on samples dried at room temperature and heat treated up to 600 °C are shown in Fig. 3. Heat treatments at room temperature, 60, and 100 °C exhibit strong 00*l* peaks at ∼12 and ∼25° 2*θ*, typical of layer-disordered birnessites. Between 200 and 400 °C, dehydration results in diminished coherence between adjacent layers resulting in substantial broadening of the 00*l* reflections. Intensity is observed in approximately the positions corresponding to T_2,2_ α-MnO_2_, though laboratory PXRD data is inconclusive. At 500 °C major differences in the phase composition become clear, with bixbyite (α-Mn_2_O_3_) and hausmannite (α-Mn_3_O_4_) becoming more crystalline with increasing temperature.

### 3.2 Raman spectroscopy

**Fig. 4:**
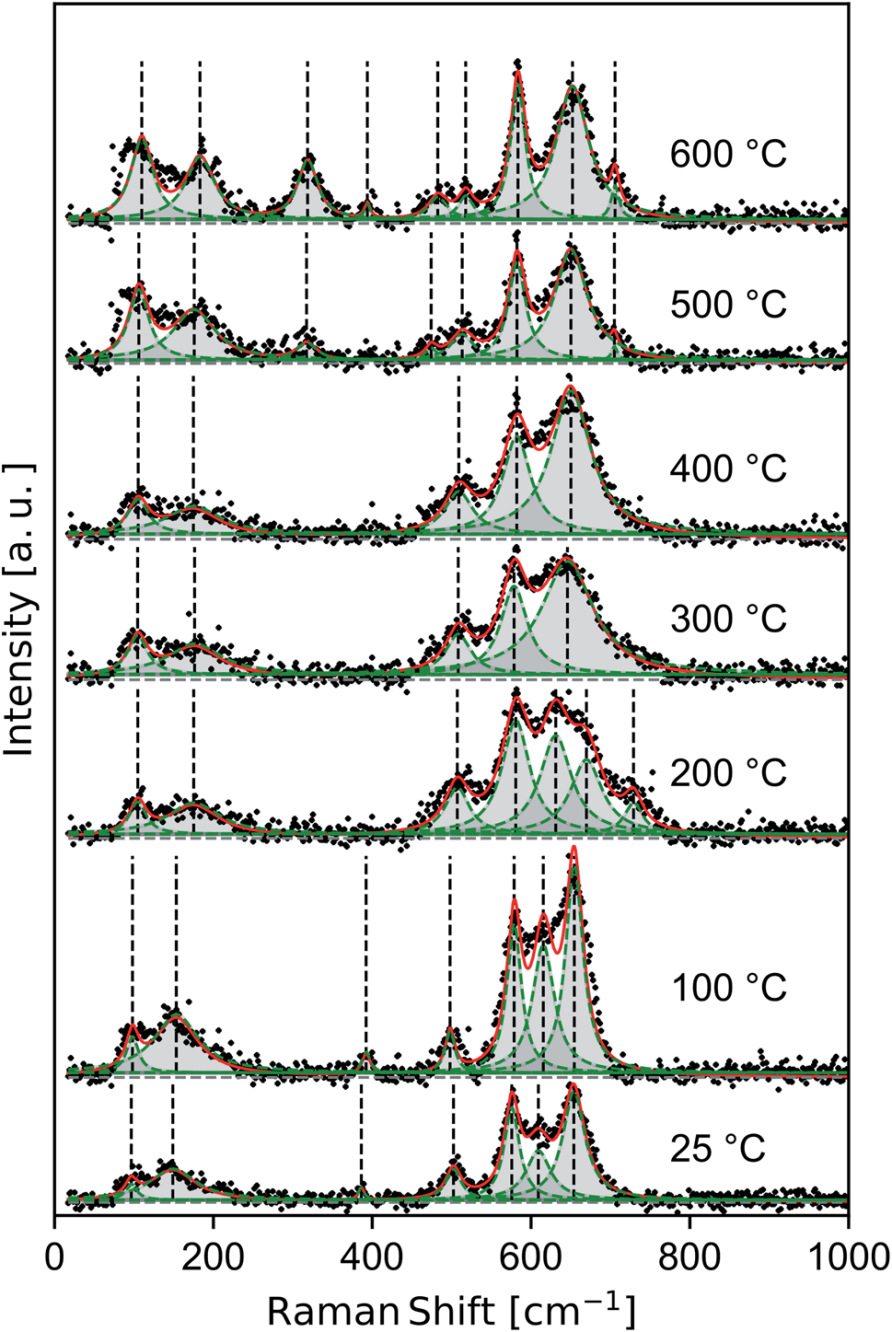
Raman spectra measured *ex situ* for δ-MnO_2_ nanosheets flocculated with HCl at pH 2. Composite (red line) and individual (green peaks) components of the fit.

Raman spectra of samples dried at room temperature and heat treated to 100–600 °C (Fig. 4) show features consistent with previous band assignments of birnessite-like and cryptomelane-like manganates,^27–29^ and in particular with birnessite of predominantly solvent-filled galleries.^53^ The band positions are tabulated (Table 1) using band assignments consistent with Julien and coworkers.^27–29^

**Table 1:** Fitted band positions of the Raman spectra

*T* [°C]	External		Band position [cm^−1^]								
			*ν* ^Bix^_2_	*ν* ^Bix^_3_	*ν* ^Bir^_3_	*ν* ^Bix^_4_	*ν* ^Bir^_4_, *ν*^Cr^_4_	*ν* ^Bir^_5_, *ν*^Cr^_5_	Unknown	*ν* ^Bix^_6_, *ν*^Cr^_6_, *ν*^Bir^_6_	*ν* ^Bix^_7_, *ν*^Bir^_7_
25	97	149	—	—	386	—	502	576	609	654	—
100	98	153	—	—	392	—	498	579	615	654	—
200	105	175	—	—	—	—	507	581	631	670	729
300	105	176	—	—	—	—	508	579	—	646	—
400	105	175	—	—	—	—	509	582	—	650	—
500	106	176	317	—	—	474	513	582	—	650	705
600	110	183	319	394	—	483	518	584	—	652	706

The spectra show a distinct set of as-yet unassigned low-wavenumber bands (*ca.* 100 and 150–185 cm^−1^) which sharpen and shift to slightly higher energy with increasing heat treatment. These low energy vibrations may constitute a pair of external or intramolecular modes,^54–56^ in contrast with the intermolecular Mn–O vibrations characteristic of higher energy phonons. Spectra in the 25–200 °C regime exhibit strong features at 385, 505, 575, and 655 cm^−1^ belonging to a hydrated acid birnessite.

Between 200–400 °C, the Raman spectra broaden and then re-sharpen into three bands around 510, 580, and 645 cm^−1^ belonging characteristically to T_2,2_ α-MnO_2_.^57^ At 500 and 600 °C new modes near 320, 395, and 706 cm^−1^ sharpen into existence, and the *ν*_4_ mode of birnessite and α-MnO_2_ near 505 cm^−1^ is seen to split into a doublet centered near 480 and 515 cm^−1^. These discontinuous changes in the observed Raman spectra are consistent with the decomposition of α-MnO_2_ into α-Mn_2_O_3_, as noted in the laboratory diffraction data.

An additional band ranging from 609 to 631 cm^−1^ persists up to 200 °C between the *ν*_4_ and *ν*_5_ birnessite modes. While this mode is inconsistent with band assignments based on an idealized *C*^3^_2h_ symmetry (*e.g. C*2/*m* birnessite),^27^ the resulting broad plateau between the dominant bands at 575 and 655 cm^−1^ is a general feature of many hydrated, protonated birnessite specimens.^27,53^ The δ-MnO_2_ floccules studied here possess ∼20 at% Ruetschi defects^35^ (*i.e. V*_Mn_ capped by Mn^3+^O_3_(OH)_3_ octahedra), introducing a number of additional manganese-bonded species and reducing the nominal symmetry of the δ-MnO_2_ layer. Alternatively, the difference between the structure of δ-MnO_2_ with extensive Ruetschi defects and T_2,2_ α-MnO_2_ is largely one of interlayer and defect ordering. The α-MnO_2_ Raman spectral features largely overlap with those of birnessite, but include an allowed Raman mode^28^ at 628 cm^−1^. While other α-MnO_2_ bands are absent, such as the strong band at 259 cm^−1^, the presence of this mode at room temperature may imply the local nucleation of tectomanganate fragments.

The internal, or higher wavenumber, modes belong generally to the Mn–O framework.^29^ As such, they are diagnostic of the degree of Mn–O polymerization in a given specimen.^26,28^ Further, the intensity ratio of various internal Raman modes reflects the ability of intercalated species to dampen Mn–O oscillations, although intercalated species exhibit no Raman-active modes themselves.^28,57,58^ The slight increase in wavenumber of the *ν*_4_ band throughout the spectra indicates a decrease in the number of edges shared per MnO_6_ octahedron,^26,28^ consistent with the introduction of triple-corner-shared (TCS) octahedra characteristic of the T_2,2_ α-MnO_2_ structure.

External Raman modes are typically associated with molecular crystals, but have been demonstrated to occur in analogous layered structures like MoS_2_ where they represent various breathing and shear modes of the layer structure.^59^ The observation of two low-energy Raman modes, which sharpen as a function of heat treatment, are distinct from spectra typically reported for bulk crystalline birnessites and α-MnO_2_. For example, Julien and Massot^29^ report no external modes in a sol–gel derived hydrated birnessite, while studies of hydrated α-MnO_2_ typically report a single mode^57,58^ near 185 cm^−1^. We tentatively interpret these low energy modes as external modes, representing shearing and breathing of few δ-MnO_2_ layers. In this work, both external bands increase measurably in wavenumber as a function of heat treatment, ranging from 97–110 and 149–183 cm^−1^, respectively. The increase in energy as a function of heat treatment and the increasing sharpness of these two bands is consistent with an enhanced crystallinity of the nanosheet ensemble, suggesting heat treatment drives increased interlayer bonding.

Finally, we note that the presence of these low-energy modes and their sensitivity to the arrangement of the δ-MnO_2_ slab as a rigid unit presents the opportunity to characterize δ-MnO_2_ nanosheet assemblies using hyperspectral Raman imaging, as has recently been performed in the case of molybdenum dichalcogenides.^60^ If the technical challenges associated with the low Raman activity of MnO_2_ can be overcome, the apparent sensitivity to degree of crystallinity and to the δ to α phase transformation may provide a spectroscopic approach to spatially resolve the layer-to-tunnel conversion of these materials with ∼1 μm resolution when using confocal Raman.

### 3.3 Powder diffraction inference

**Fig. 5:**
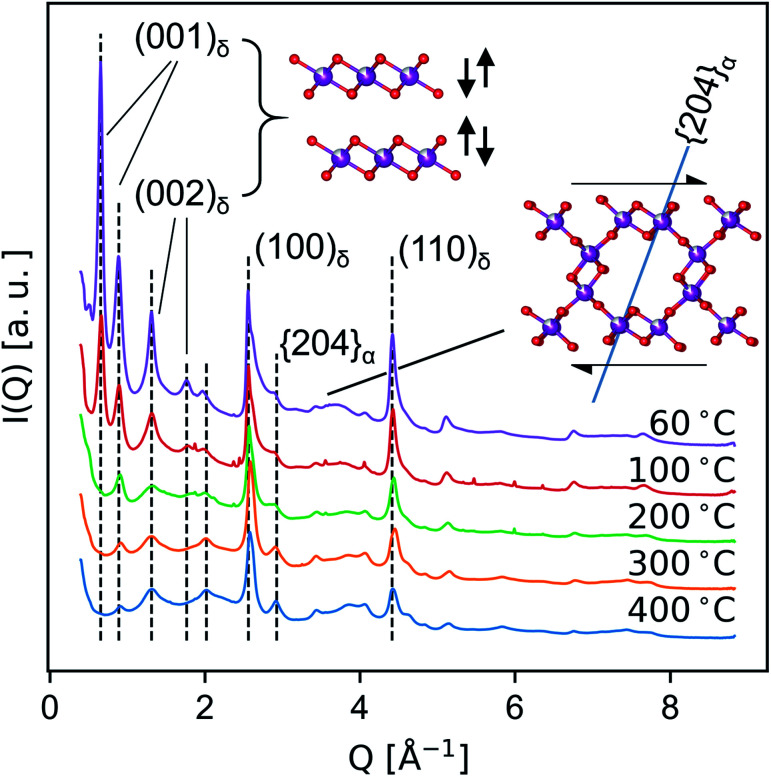
Evolution of *ex situ* SXRD profiles as a consequence of heat treatment, highlighting the decoupled sensitivity of different *hkl* to layer spacing (left) and lateral ordering (right). The (20̄4)_α_ plane is drawn for clarity (right).

**Fig. 6:**
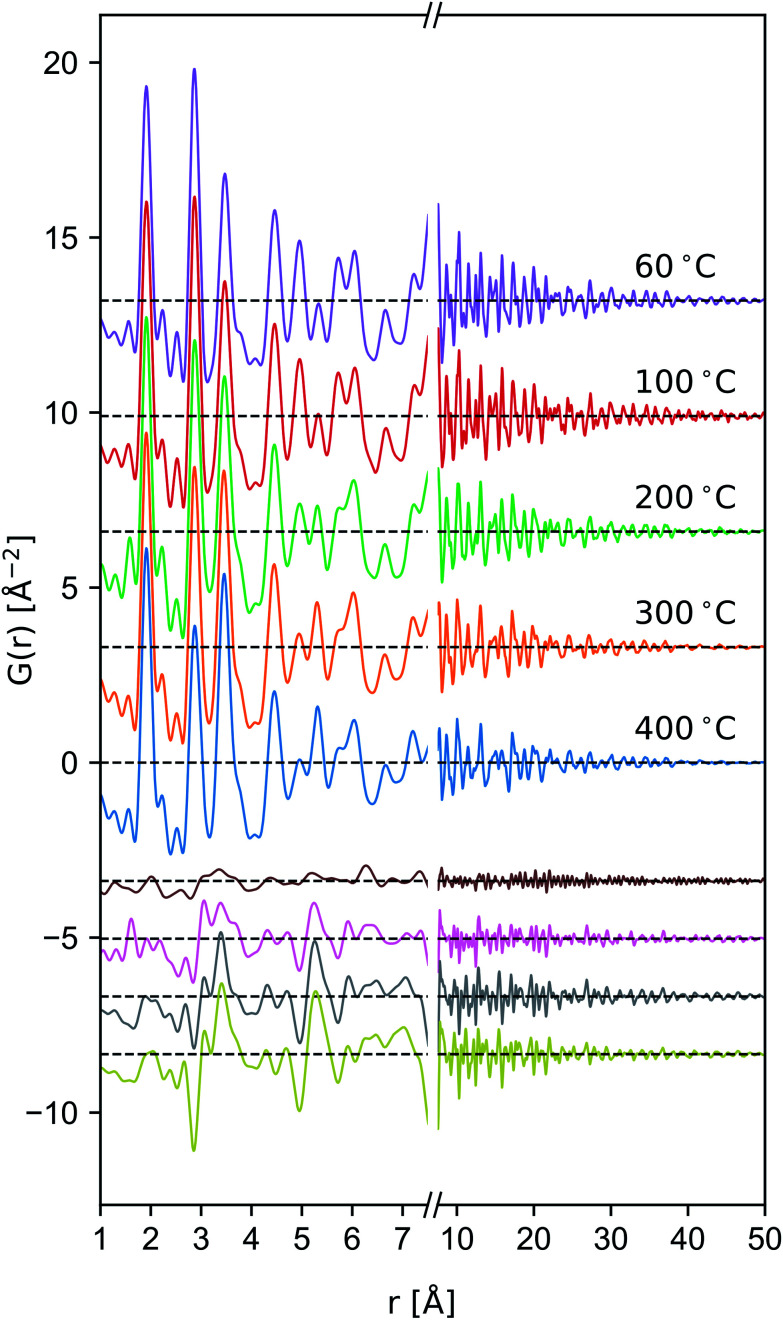
Comparison of the reduced PDF of δ-MnO_2_ nanosheet floccs heated to the indicated temperatures. Difference patterns (offset, below) are computed as *G*(*r*, *T*) − *G*(*r*, *T* = 60 °C) from *T* = 100 to 400 °C in descending order.

Medium-resolution synchrotron powder diffraction and X-ray atomic PDF data (Fig. 5 and 6) were used to investigate the phase evolution of the δ-MnO_2_ nanosheet floccules in more detail. In layer disordered ensembles, diffraction profiles typically exhibit sharp in-plane *hk*0 and basal 00*l* reflections while diffracting planes mixing *h*-, *k*- and *l*-indices become severely broadened.^61^ The observed set of diffraction profiles (Fig. 5) exhibit two relatively sharp, asymmetric features near 2.5 and 4.5 Å, which correspond to the (100) and (110) lattice planes of the trigonal (*a* = *b* = 2.85 Å, *γ* = 120°) δ-MnO_2_ unit cell. An irrational basal series is observed below 200 °C, indicating a mixture of layer types with approximately 7 and 9.5 Å layer spacings, typical of differing interlayer hydration states.^62,63^ (The synthesis method employed will yield either 7 and 9.5 Å layer spacings, or all 7 Å layer spacings, depending on the number of wash cycles.) The loss of intensity in the 00*l* diffraction profile correlates to the dehydration of the manganate interlayer structure by *ca.* 220 °C, consistent with typical thermogravimetric analysis (ESI†). This loss of intensity is attributable to both a change in periodic electron density as well as a decreased coherence length in the *c* column.

The region between the (100)_δ_ and (110)_δ_ features exhibits a complex and severely broadened intensity distribution, indicative of substantial layer disorder. The existence of distinct shapes in this data segment which sharpen systematically with temperature imply the flocculated nanosheets have limited but finite lateral structural coherence rather than completely turbostratic character.^61,64^

In the T_2,2_ α-MnO_2_ structure, the tunnel runs parallel to the c crystallographic direction, while the tunnel walls resembling δ-MnO_2_ fragments align with the (110)_α_ set of planes (*i.e.* (110)_α_ is isomorphic with (001)_δ_.) As the material is heated, the shoulder on the first δ-MnO_2_*hk*0 band is seen to resolve into two distinct peaks, which can be indexed as the overlapped (031)_α_ and (040)_α_, and the (20̄4)_α_ planes, respectively. The (20̄4)_α_ plane forms an angle of approximately 71.4° with the (110)_α_ plane (*i.e.* the birnessite basal plane), and thus is diagnostic of the degree of interlayer ordering (inset, Fig. 5). The sharpening of the (20̄4)_α_ diffraction feature while the Bragg peaks mixing *l*-indices remain broadened suggests that at temperatures below 400 °C the transformation proceeds by first ordering adjacent defective δ-MnO_2_ layers.

This result is consistent with the recent aberration-corrected scanning transmission electron microscopy (STEM) analysis of the layer-tunnel conversion in a Mg_*x*_MnO_2_ buserite-like material reported by Yuan *et al.*,^65^ which highlights the nucleation of tunnel fragments by both alignment of displaced intralayer Mn^3+^, and short-range lateral diffusion of interlayer Mn-species. We note the tunnel conversion revealed by STEM shows a plurality of tunnel polymorphs caused locally by nucleation of various width tunnels, and at longer length scale by intersecting tunnel-nucleated domains (*i.e.* growth faults).^65,66^ Analogously, the initial intergrowth of various T_*m*,*n*_ fragments is a likely contributor to the diffuse X-ray scattering bands present in the data.

Fitting powder diffraction data of layer disordered manganates is notoriously challenging. A suitable model must account for a number of factors, including (i) the point defect content and relaxation of the layer motif, (ii) the position fluctuation of adjacent layers, (iii) the influence of highly anisotropic particle shapes and sizes, and (iv) the influence of bending and other distortions. This has been done rigorously for δ-MnO_2_ nanosheet ensembles and other 2D materials on relatively few occasions,^64,67–70^ and is not pursued in this work. Alternatively, PDF analysis is performed to provide insight into the local and intermediate range structure evolution, detailed below.

### 3.4 X-ray PDF analysis

#### 3.4.1 Model-free inferences

Qualitatively, the *ex situ* X-ray PDF data exhibit the characteristic attenuation of nanoscale materials, with pair correlations largely diminished by 50 Å (Fig. 6). In the defective δ-MnO_2_ structure, in-layer sites will be denoted Mn^L^ and O^L^ whilst out-of-layer sites will be denoted Mn^IL^ and O^IL^. The first three correlation peaks in the ideal δ-MnO_2_ structure belong to the first MnO_6_ correlation polyhedra (∼1.9 Å), the first Mn^L^⋯Mn^L^ and O^L^⋯O^L^ correlations (∼2.85 Å), and the second nearest Mn^L^⋯O^L^ correlation (∼3.45 Å). The defective structure introduces additional correlations at ∼2.2 Å due to Jahn–Teller distortion of trivalent Mn^L^, as well as introducing Mn^L^⋯Mn^IL^ correlations at ∼3.45, ∼5.32, 6.04, ∼7.81 Å, and beyond. Consequently, the relative amplitude of the 2.85 Å and 3.45 Å peaks vary inversely and are diagnostic of the number density of Ruetschi defects in a δ-MnO_2_ sheet.

The Ruetschi defect forms a TCS arrangement with the remaining in-plane manganese vacancy, similar to the structural motif of the tunnel structured MnO_2_ polytypes. It has been hypothesized that this structural similarity causes the vacancy-adsorbed Mn^3+^ sites to lower the activation barrier for tectomanganate transformations.^32^ This interlayer condensation transformation mechanism essentially requires the ordered arrangement of vacancy-adsorbed Mn^3+^ between adjacent defective δ-MnO_2_ layers, and the elimination of chemisorbed water species to create new bridging Mn–O–Mn bonds (Fig. 1(d)).^31,32^

Tectomanganate conversion as the material is heated and dehydrated is substantiated by the reduction of Mn^L^⋯Mn^L^ (2.85, 4.95 Å) and reciprocal increase of Mn^L^⋯Mn^IL^ (3.45, 5.32 Å) correlations, appearing clearly in the difference PDF (Fig. 6). Other features, like the discontinuous change in the small ∼2.5 Å correlation peak at 200 °C may be attributable to the Mn⋯O coordination of the TCS MnO_6_ arrangement, but exhibit less sensitivity owing to the relative X-ray scattering power of oxygen.

To semi-quantitatively estimate the relative concentration of in-plane and TCS out-of-plane Mn sites, model-free analysis on the region between 1.4–4.7 Å was performed by fitting a sinc function, linear baseline, and set of constrained Gaussian peaks. The sinc function was included in the model to approximate termination ripples. The peak width of the nearest-neighbor Mn–O correlation was allowed to vary independently to account for correlated motion. Meanwhile the remaining peak widths were constrained to a single value. Fitting was carried out in the Python package Lmfit,^71^ and computation and propagation of ±1*σ* confidence intervals were calculated using Emcee^72^ and Uncertainties,^73^ respectively. Typical pattern-weighted fit residual (*R*_wp_) was on the order of ∼20% (fits presented in ESI†).

Occupancies were calculated by computing the ratio of characteristic peak amplitudes *A*_site_1_⋯site_2__, according to the equations9
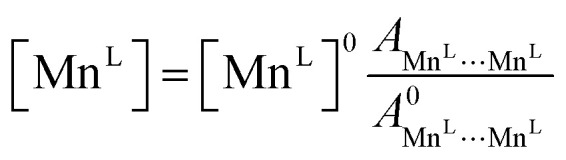
10
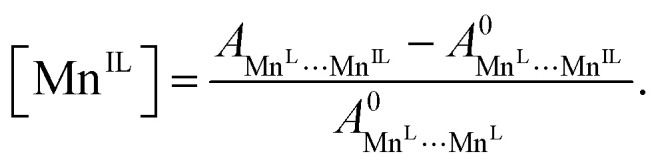
Here, it is assumed that the as-synthesized parent compound (*A*^0^) has negligible equilibrium Ruetschi defect concentration [Mn^IL^], permitting normalization of the changing concentration of Mn-sites against the parent compound correlation peaks. In each case, the refined Gaussian amplitudes were normalized by the nearest-neighbor Mn⋯O correlation peak, assuming that all Mn exist in octahedral coordination. The resulting extracted site occupancies are reported in Table 2.

**Table 2:** Occupancies extracted from model-free analysis of the 1.4–4.7 Å region of the atomic PDF

	[Mn]	[Mn^IL^]	Sum
Parent	1.0	0.0	1.0
60 °C	0.74(4)	0.19(4)	0.93(7)
100 °C	0.72(4)	0.21(4)	0.93(7)
200 °C	0.71(4)	0.28(4)	0.99(8)
300 °C	0.67(4)	0.32(4)	0.99(7)
400 °C	0.58(4)	0.35(5)	0.93(7)

For context, we note δ-MnO_2_ nanosheet floccs produced in acidic environments typically possess 10–20 at% in-plane manganese vacancies,^70,74^ where 1/7 [*V*_Mn_] represents the limit for which neighboring *V*_Mn_ are completely screened by occupied Mn^L^ sites. Meanwhile T_2,2_ MnO_2_ contain ordered arrays of 1 in 3 vacant Mn columns in comparison to the ideal δ-MnO_2_ motif.

#### 3.4.2 Local structure analysis

**Fig. 7:**
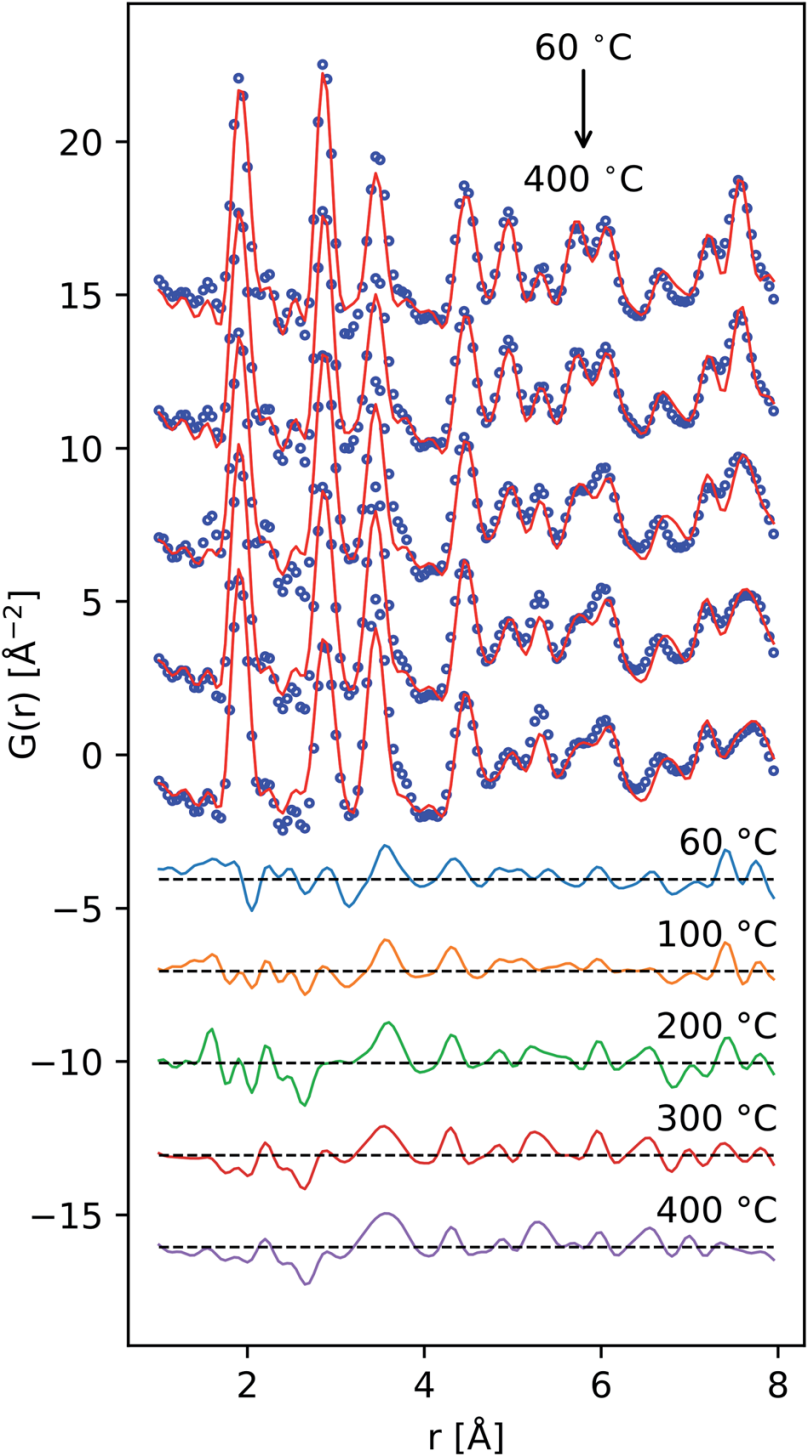
PDF data fit using a simple mixture of chalcophanite-structured MnO_2_ and α-MnO_2_ (9 parameters) reproduces many of the major low-*r* features across the temperature series.

While convenient, the preceding model-free analysis is limited by the assumption that the contributions to individual correlation peaks are completely resolved, which is certainly flawed. Under the hypothesis that the defective δ-MnO_2_ floccs are converted to α-MnO_2_, as suggested by the Raman spectra and powder diffraction profiles, a simple mechanical mixture of these two phases was fit against the first 8 Å of PDF data (Fig. 7).

The defective δ-MnO_2_ layer was represented by a chalcophanite-structured layer model, analogous to the work of Liu *et al.*^70^ This model fixes the concentration of vacant Mn^L^ sites at 1/7, assuming all vacancies are coordinated by occupied Mn^L^O_6_. The doubly capped in-plane vacancy sites (*i.e.*Fig. 1(b)) eliminate direct Mn^L^⋯Mn^IL^ correlation which are unlikely, and additionally provide a Mn^IL^⋯Mn^IL^ correlation at ∼4.15 Å.

Model parameterization was pared down to a minimal set by constraining the magnitude of the chalcophanite (*a*_chal_ and *b*_chal_) and α-MnO_2_ (*a*_α_ and *b*_α_) lattice vectors by their geometric relationship to the trigonal birnessite lattice vector *a*_trig_, or equivalently to the average Mn⋯Mn pair distance. By geometric arguments,11
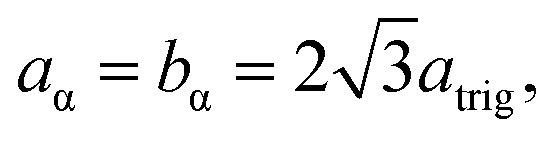

12 *c*_α_ = *a*_trig_,

and13
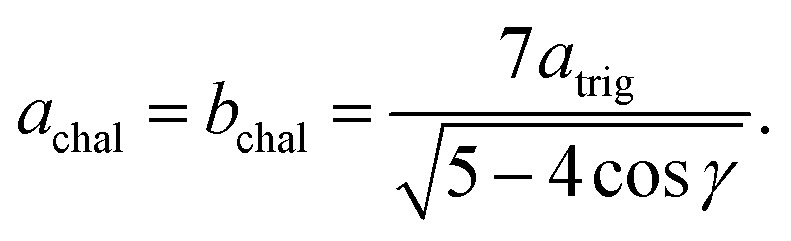


Both structures were constrained to share the correlated atomic motion^75^ parameter *δ*_2_. To represent the layer disorder, the chalcophanite model was assigned anisotropic displacement parameters (ADP) *B*_11_ = *B*_22_ ≠ *B*_33_. Oxygen ADP values were constrained simply as twice the value of the manganese sites. The remaining parameters are summarized in Table 3.

**Table 3:** Parameters fitted over 1–8 Å of the atomic PDF data

	60 °C	100 °C	200 °C	300 °C	400 °C
**Global**					
*a*_trig_ [Å]	2.847(4)	2.848(4)	2.850(4)	2.843(4)	2.847(4)
*δ*_2_ [Å^−2^]	3.25(10)	3.17(9)	2.78(20)	2.70(18)	2.59(20)

**Chalcophanite structure**					
Scale	1.86(10)	1.85(10)	1.77(15)	1.59(14)	1.39(15)
*c* [Å]	7.66(7)	7.29(5)	7.25(5)	7.25(1)	7.25(3)
*γ* [°]	119.5(2)	119.4(2)	119.3(2)	119.0(2)	118.9(3)
*B*_11_ [Å^2^]	0.12(3)	0.15(3)	0.28(7)	0.30(8)	0.49(13)
*B*_33_ [Å^2^]	17(3)	13(2)	13(4)	5.4(1.7)	2.1(1.0)

**α-MnO**_**2**_ **structure**					
Scale	0.50(7)	0.40(7)	0.63(10)	0.69(9)	0.80(9)
*B*_iso_ [Å^2^]	0.14(5)	0.11(5)	0.16(6)	0.18(5)	0.22(5)

**Estimated Mn** ^**L**^ **occupancy**					
Occ. Mn^L^ [−]	0.816(5)	0.823(5)	0.807(7)	0.800(6)	0.788(7)
*R*_wp_ [%]	23.8	23.0	30.5	26.1	27.6

In the lowest temperature data set, the fit residual approaches the value obtained in the model-free analysis, near 20 at%. Considering this as a baseline, it is clear this simple model misses some components of the PDF, with *R*_wp_ reaching 30.5% in the 200 °C data set. However, the relative peak amplitudes are largely reproduced.

There are two notable trends in the refined parameters. First, the estimated δ-MnO_2_ : α-MnO_2_ ratio decreased from about 80 : 20 for material dried at 60 °C, to about 60 : 40 for materials heated to 400 °C on a volume basis. Second, the refined value of *B*_33_, approximating the position fluctuation of adjacent layers, diminished from ∼17 Å^2^ to ∼2 Å^2^, reflecting a substantial enhancement in the interlayer ordering.

Considering the α-MnO_2_ structure to be comprised of organized δ-MnO_2_ layers possessing 1/3 vacant Mn^L^ sites doubly capped by Mn^IL^O_6_ units, the average Mn^L^ occupancy in context of the δ-MnO_2_ motif may be construed as14
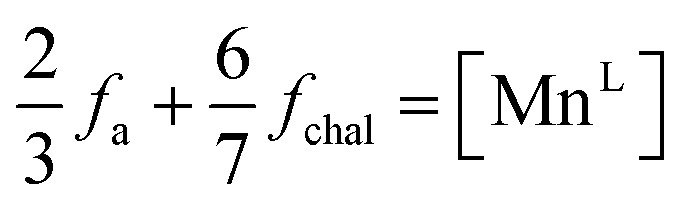


These values (Table 3) are generally larger than the values estimated by model-free fitting, presumably due to the ability to correctly account for overlapped pair correlations. The same trend is displayed, with an initial defect population near 18 at% increasing above 100 °C to ∼22 at%.

#### 3.4.3 Intermediate range analysis

The interlayer condensation reaction requires both the formation of vacancy-adsorbed Mn^3+^O_6_ units as well as the ordering of these units between adjacent δ-MnO_2_ layers. To examine the local structure influence of intralayer ordering, the pattern-weighted PDF fit residual was computed over the interval 1–25 Å. The layer–layer correlation was examined using 18-layer supercell stacking models^76^ (*ca.* 500 atoms per cell) spanning the translational stacking vector space (±1*R*_*x*_, *R*_*y*_) on a 100 × 100 mesh. The layer definition was taken as the chalcophanite model, with a doubly capped layer vacancy per unit cell (1/7*V*_Mn_). The scale of the computed PDF was constrained such that the amplitude of the nearest neighbor correlation peak equaled that of the observed PDF. The lattice parameters and nanoparticle shape envelope were optimized initially and subsequently fixed for the array of computations.

**Fig. 8:**
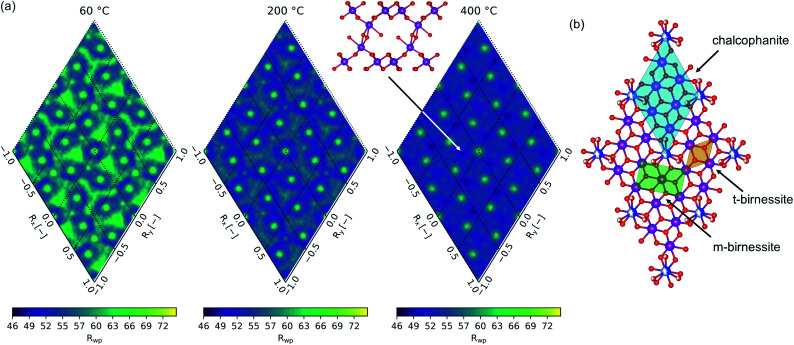
PDF residuals computed from 1–25 Å and mapped across ±1 *R*_*x*,*y*_ show likely positions of the adjacent layer (a). Projection in the (001) plane (b) demonstrates the relationship of the maps in (a) to the δ-MnO_2_ layer motif.

The computed fit-maps projected in the (001) plane for material heated to 60, 200, and 400 °C are presented in Fig. 8(a), with the relationship to the δ-MnO_2_ motif illustrated in Fig. 8(b). The 25 Å PDF residual reveals a nuanced preference for the stacking of adjacent layers, with the direct superposition of TCS Mn^IL^ sites least likely (highest residual), and a number of other apparently degenerate configurations. In the 400 °C specimen, the set of degenerate probable states (lowest residuals) resolve from more fluid, continuous space into apparent discrete states, consistent with the increased layer order evident in the powder diffraction profiles, and in the refined anisotropic thermal parameters *B*_11_ and *B*_33_ (Table 3). For reference, the mineral chalcophanite crystallizes in the minima at *R*_*x*,*y*_ = (2/3*a*, −2/3*b*) with respect to the chalcophanite lattice vectors.

Inspection of the interlayer gallery in the three-fold set of degenerate states identifiable around the origin (*R*_*x*_, *R*_*y*_ = 0, 0) is roughly consistent with the interlayer ordering mechanism proposed by Lanson *et al.*^77^ for H^+^ exchanged birnessites. Lanson and coworkers posited that adjacent layer position is determined by the competition between hydrogen bonding from vacancy-capping MnO_3_(OH)_3_ to the adjacent δ-MnO_2_ layer and the electrostatic repulsion of the negatively charged δ-MnO_2_ layers. Enhanced hydrogen bonding results in orthogonal stacking of adjacent δ-MnO_2_ layers, minimizing the distance between (OH)^IL^⋯O^L^ sites but resulting in the closest Mn⋯Mn separation. Where electrostatic repulsion is dominant, adjacent layers tend to be offset with respect to the cation sublattice.

The preference of adjacent layers shown here suggests layers are offset into three-fold degenerate positions, reminiscent of the layer symmetry. Further, visualizing the molecular fragments formed in these states highlights what appears as the onset of interlayer cross linking *via* the formation of edge shared MnO_6_ fragments. This strongly resembles a T_2,2_ α-MnO_2_ tunnel fragment sheared perpendicular to the [110̄] vector.

## 4 Electrochemical behavior

**Fig. 9:**
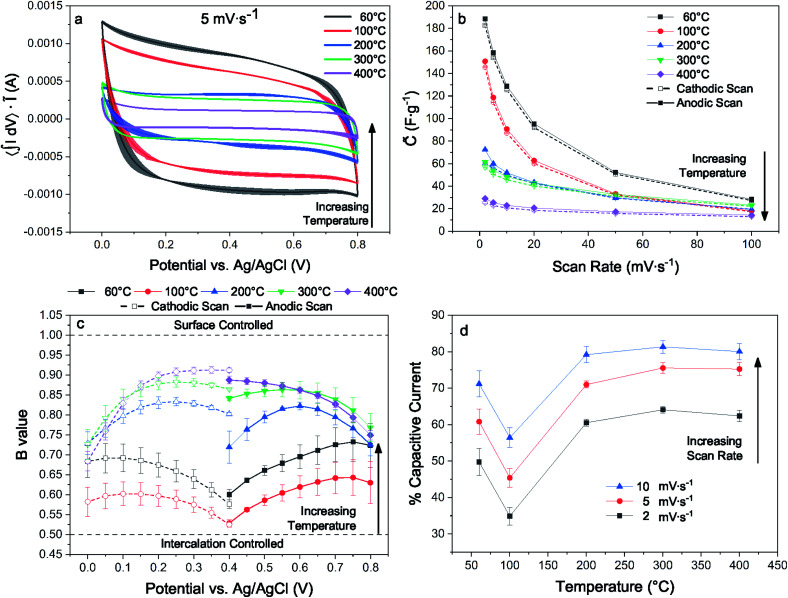
Typical CV loops scaled to area acquired at a scan rate of 5 mV s^−1^ (a), dependence of specific capacitance upon scan rate for cathodic and anodic sweeps (b), *b*-value determination by linear regression of cathodic and anodic sweeps (c), percentage based surface capacitive contribution to current from 2–10 mV s^−1^ (d).

**Fig. 10:**
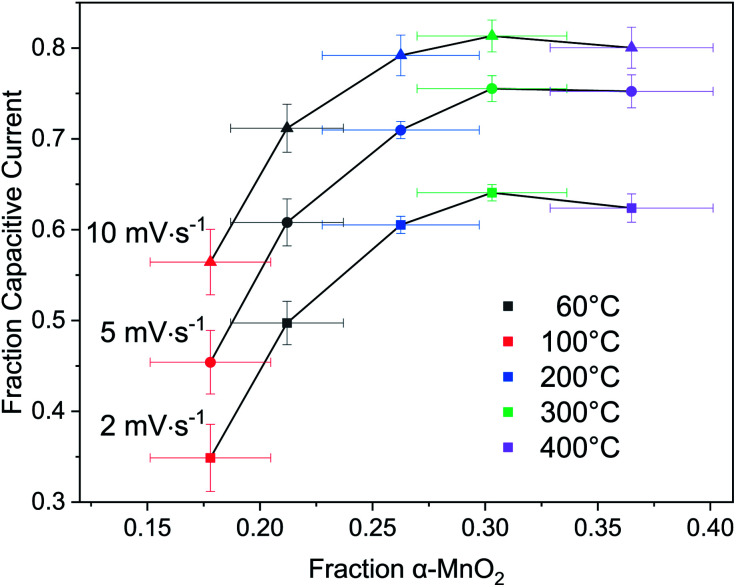
Correlation of fraction EDL capacitive current to α-MnO_2_ phase fraction.

For electrodes with identical preparation, dehydration and interlayer condensation of the layer disordered δ-MnO_2_ nanosheet cluster has a substantial influence on pseudocapacitive performance observed by cyclic voltammetry (CV). This is immediately evident from the measured voltammograms, particularly at lower sweep rates (5 mV s^−1^, Fig. 9(a); 2 and 10–100 mV s^−1^, ESI†). Across the temperature series, increased heat treatment leads to a diminished capacitive current.

Previous work on polymorphs of MnO_2_ suggests that even nanoscale partial conversion from the layer to the tunnel form should be reflected in the electrochemical response. Devaraj and Munichandraiah^16^ showed that the specific capacitance of the α and δ polymorphs with are similar when prepared with similar SSA, but that the tunnel form suffers from diffusional limitations at high scan rates. Additional work by Ghodbane and coworkers^78^ showed the response of the parent crystal structures to ion intercalation and deintercalation, and Pan and coworkers^79^ later showed the differences between the layered and tunnel forms in terms of kinetics and capacity fade. Indeed, even direct observations of the layer to tunnel conversion under electrochemical cycling has been reported for Zn battery cathodes.^17^

The electrodes bearing MnO_2_ heated to 200, 300, and 400 °C increasingly approach a square trace, characteristic of kinetically unencumbered reversible processes like electrical double layer (EDL) capacitance. The electrodes heated to 60 and 100 °C show markedly different voltammograms, including an asymmetry in the anodic and cathodic sweeps. This is characteristic of a diffusionally controlled reversible charge storage process, as the cathodic and anodic processes require different potentials to establish concentration gradients in the boundary diffusion layer leading to identical diffusional currents.^80^ We interpret the differences between the high and low temperature samples, which have similar surface areas, as evidence for structure changes, as expected from earlier work demonstrating kinetic limitations for ion intercalation into cryptomelane tunnels.

A kinetic analysis of the cyclic voltammetry was performed to further qualify the changes in electrochemical charge storage mechanism between electrodes. The *b*-value was determined for the range of scan rates 2–100 mV s^−1^ in the voltage window from 0.0–0.4 and 0.4–0.7 V for the cathodic and anodic sweeps, respectively (Fig. 9(c)). Linear regressions of the *b*-value can be found in the ESI.† These values were generally intermediate in nature, trending towards larger values with increased heat treatment. The notable exception is for the lowest temperature treatment where the trend is reversed, indicating an electrode that is less kinetically inhibited yet exhibits substantially greater capacitance.

The contributions of Na^+^ insertion and surface charge storage mechanisms can be quantified by solving eqn (13) for the slope and intercept, corresponding respectively to the EDL current and diffusive current. Fig. 9(d) shows the percent capacitive current calculated for each temperature treatment and for 2–10 mV s^−1^. The samples all show an expectedly increasing capacitive character with increased scan rate. Analogous to the *b*-value analysis, the sample treated to 100 °C shows the greatest degree of diffusionally limited current, and samples heated to 100 °C and above show an increasingly capacitive character.

These analyses are reflected in the convergence of the quasi-specific capacitance (Fig. 9(b)) towards ∼30 F g^−1^ with increasing sweep rate, which is likely the limiting EDL capacitance corresponding to the specific surface area of this material. The quasi-specific capacitance diverges at lower scan rates, with the 400 °C sample increasing by a factor of ∼2, while the 60 °C sample increases by a factor of ∼6.

Returning to the hypothesis that the fraction of tectomanganate fragments in the MnO_2_ structure are the key kinetically limiting factor in the Na^+^ intercalative pseudocapacitance, the correlation between the calculated fraction of capacitive current and the refined fraction of α-MnO_2_ was examined (Fig. 10). In the samples heated up to 200 °C, coincident with the primary dehydration event of the MnO_2_ (ESI†), there is a clear positive correlation between the structure evolution and fraction EDL capacitance. However, above 200 °C the change in charge storage mechanism becomes insensitive to the increasingly 3D MnO_2_ framework.

This result suggests one of two possibilities: (i) the change in intercalative pseudocapacitance is driven by the hydration state, not the formation of tectomanganate fragments, or (ii) the intercalative Na^+^ diffusion reaches a type of percolation threshold at around 25–30% tectomanganate formation. In either case, it must be concluded that the intermediate behavior of MnO_2_ electrodes formed between the δ- and α-MnO_2_ end members of this phase space are not described by a simple rule of mixtures.

Future work will examine the role of electrochemical strain and its accommodation in these disordered nanosheet floccs, following recent reports on the function of structural water in hydrated tungstates during electrochemical H^+^ insertion.^81^ Large point defect concentrations and local formation of bridging tectomanganate fragments influences the compliance of the manganate and its ability to accommodate electrochemical strain, with implications for electrode capacity and cyclability, which we will report separately.

## 5 Summary of findings

The key findings for the δ-MnO_2_ floccs heated between 60 and 400 °C are summarized as follow:

(1) The initial structure is comprised of a ∼7 Å layer spacing corresponding to an acid birnessite analog, and a ∼9.5 Å layer spacing corresponding to an acid buserite analog.

(2) The initial structure has moderate coherence orthogonal to the δ-MnO_2_ plane, but substantial lateral layer position disorder.

(3) Heating promotes increased registry between adjacent layers, as revealed by Raman spectroscopy, X-ray powder diffraction, and PDF analyses. The process is not spatially uniform, resulting initially in decreased average structural coherence, consistent with STEM observations of others.^65^

(4) The PDF data are not severely attenuated as a function of temperature (Fig. 6), suggesting lateral coherence of the atomic structure is maintained which is consistent with an interlayer condensation mechanism.^32^

(5) Increasing the 3D polymerization of the MnO_6_ framework up to ∼30% in these nanostructured floccs increases the kinetic barrier to Na intercalation, as indicated by the rate dependence of the electrode specific capacitance.

(6) Above 400 °C the material begins to transform from the metastable defective δ-MnO_2_ flocc towards the thermodynamically stable α-Mn_2_O_3_ and α-Mn_3_O_4_ phases. The initial density gradients of the meso/macroporous δ-MnO_2_ flocc are transferred to the new phase, resulting in coral-like microstructures with dense α-Mn_2_O_3_ nodules.

We note that the population of interlayer Mn^3+^ and intralayer Mn-vacancies is controlled in the starting material by equilibration of δ-MnO_2_ floccs at a particular pH.^74^ Thus, it may be feasible to influence the phase composition at a given temperature and state of dehydration by controlling the pH and electrolyte chemistry in the material synthesis and electrode fabrication.

## 6 Conclusions

The phase transformations that occur in δ-MnO_2_ nanosheet floccules are highly dependent on their defect content and interlayer contents. In this work, alkali-free nanosheet floccules with an initial ∼20 at% Ruetschi defect population are demonstrated to convert towards a severely disordered α-MnO_2_ upon dehydration, consistent with an interlayer condensation mechanism. Preservation of macro- and mesopores defined by the initial nanosheet lateral dimensions and flocculation procedure, and the formation of 1D tunnel micropores evolved during the layer condensation reaction, thus yield hierarchical porosity.

The presence of the Raman mode near 630 cm^−1^ as well as analysis of X-ray atomic PDF data suggest that this interlayer condensation mechanism produces local fragments of tectomanganate as low as 60 °C, despite presentation of a powder diffraction profile consistent with a layer disordered birnessite. This nanoscale-disordered three-dimensional connectivity has important implications for the intercalative diffusion of various interlayer species, and opens a new avenue to synthesize solids with fixed micropores (tunnel dimensions) and controlled meso- and macropore sizes. As demonstrated here, dehydration and increasing tectomanganate formation results in diminished intercalative pseudocapacitance, consistent with an increased diffusional barrier for Na ion insertion.

The temperatures and conditions for which this transformation is relevant has implication for the processing and assembly of manganate-based capacitive devices, as well as for any related technologies reliant on this class of materials. It is apparent that processes preserving a phyllomanganate structure with lower interlayer atomic density help to maintain kinetically accessible Mn redox sites, increasing the intercalative pseudocapacitance and energy density of the resulting electrode. We expect that similar structure–property links may be found where the defect content critically controls the catalytic activity, for example.

## Conflicts of interest

There are no conflicts to declare.

## Supplementary Material

RA-010-C9RA08432K-s001
