# Supplementary material for: Hierarchical porosity via layer-tunnel conversion of macroporous δ-MnO2 nanosheet assemblies
Source: RSC Adv. 2020 Jan 8;10(3):1484–97. doi: 10.1039/c9ra08432k (PMC9047169; doi:10.1039/c9ra08432k)
Supplement: RA-010-C9RA08432K-s001 [file RA-010-C9RA08432K-s001.pdf]

Electronic Supplementary Material (ESI) for RSC Advances.
This journal is © The Royal Society of Chemistry 2019

Hierarchical porosity via layer-tunnel conversion of macroporous δ-MnO_2 nanosheet assemblies

Peter C. Metz,[a‡] Alec C. Ladonis,[‡] Peng Gao,[b] Trevyn Hey, and Scott T. Misture*

NYS College of Ceramics, Alfred University, Alfred, NY 14802, USA.
** Email: misture@alfred.edu*
‡ These authors contributed equally to this work.
Present address:
[a] Spallation Neutron Source, Oak Ridge National Laboratory, Oak Ridge, TN 37830, USA.
[b] College of Materials Science and Engineering, Hunan University, Chengsha, Hunan Province 410082, China.

THERMOGRAVIMETIC ANALYSIS

Figure S1 shows the typical dehydration behavior of a δ-MnO_2 nanosheet floccule powder, with primary dehydration due to loss of physisorbed water by 100 °C, and removal of structural water by ~220 °C. Continued mass loss is associated with the phase transformations to α-MnO_2 and α-Mn_2O_3 at higher temperature.

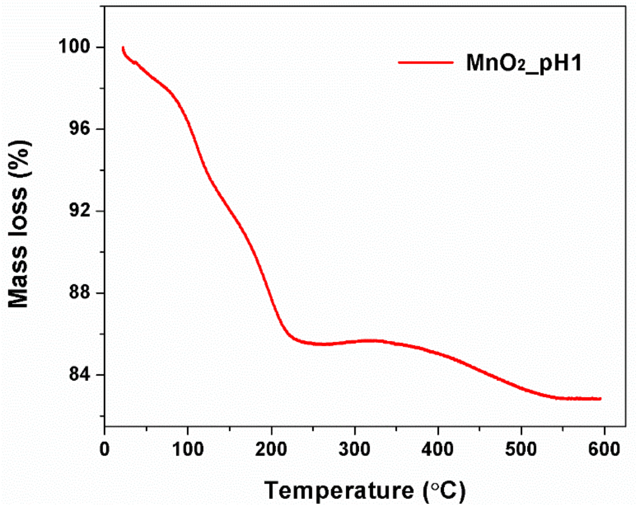

Figure S1. Mass loss signal of a flocculated MnO_2 nanosheets powder equilibrated at pH 1 in HCl.

PARENT VS. ION-EXCHANGED POWDER XRD

Laboratory X-ray powder diffraction (PXRD) data of parent K_xMnO_2 and ion exchanged powder collected in Bragg-Brentano (reflection) geometry show strong 00l reflections, in part due to plate-like powders and preferred orientation, and in part due to the diffraction peak structure factors. Upon protonation, the d-spacing of the 001 basal reflection increases by 0.28Å, indicating an increased repulsion of the partially negatively charged Mn(III, IV)O_2 sheets due to removal of the charge balancing K^+ ions in the interlayer gallery.

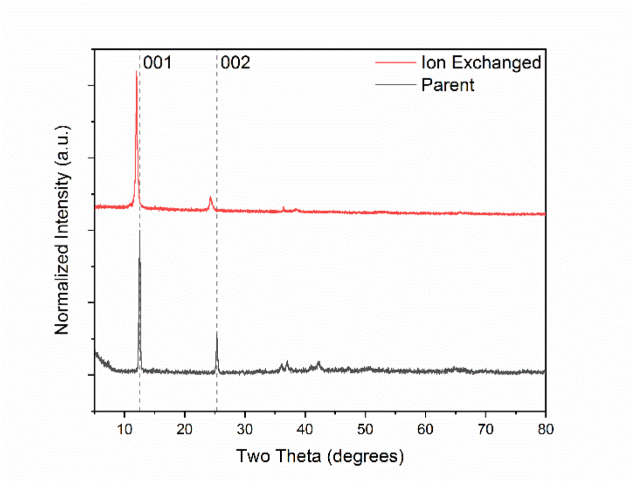

Figure S2. Typical laboratory PXRD data for parent potassium birnessite (K_xMnO_2) and the protonated derivative H_xMnO_2.

NITROGEN ADSORPTION ISOTHERMS

Specific surface area (SSA) and pore characteristics were investigated using isothermal nitrogen adsorption studies. Data were measured using a Micromeritics TriStar II. Due to the metastable nature of the flocculated MnO_2 nanosheet material, the room temperature sample (RT) was degassed for 3 days at ambient temperature under vacuum. The RT degassed sample was heated to 300 °C directly thereafter and returned to the BET instrument for data acquisition.

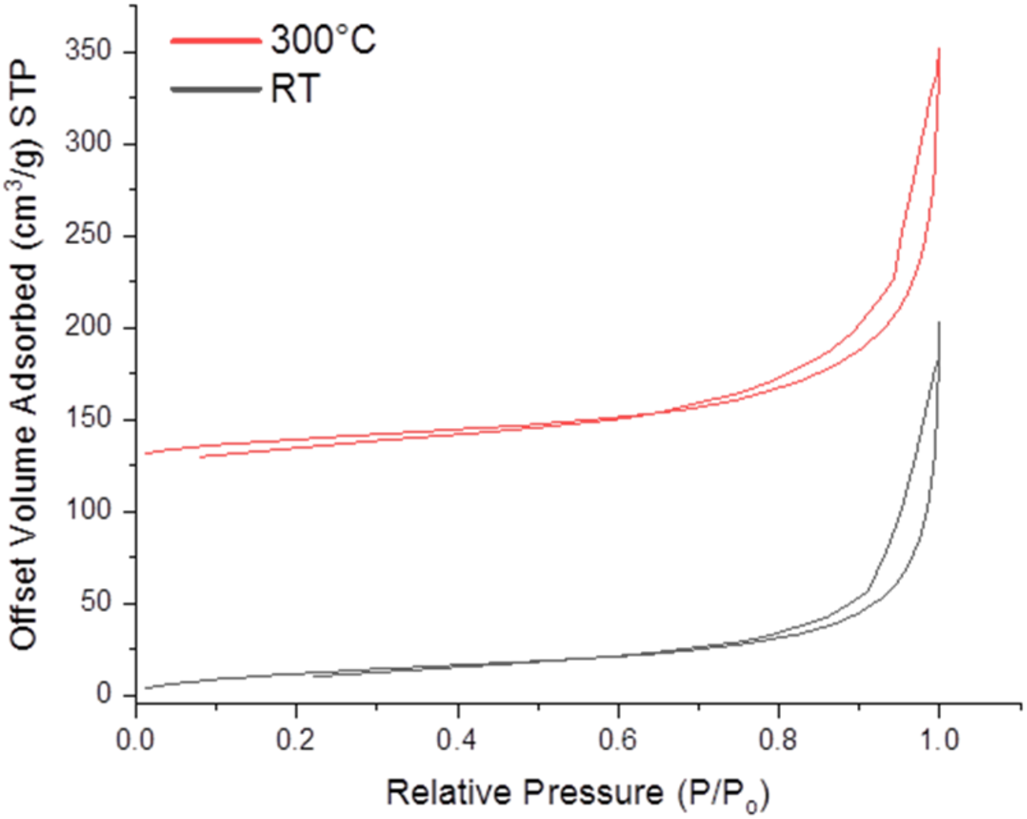

Figure S3. Nitrogen adsorption isotherms for δ-MnO_2 floccs dried at room temperature (black) and heat-treated to 300 °C (offset, red). SSA is ~80 $m^2 \cdot g^{-1}$ in both samples.

MODEL-FREE FITTING OF THE δ-MnO_2 LOCAL STRUCTURE

Estimates of in-plane (Mn^{L}) and out-of-plane (Mn^{IL}) manganese occupancies were obtained by comparing peak areas in the parent K_xMnO_2 (nominally ppm defect concentrations) and derived nanosheet ensembles. Peak areas were extracted by fitting Gaussians to the low-r region of the atomic PDF. The details of the fitting process are presented in the main text. The fits are plotted below for inspection of fit quality.

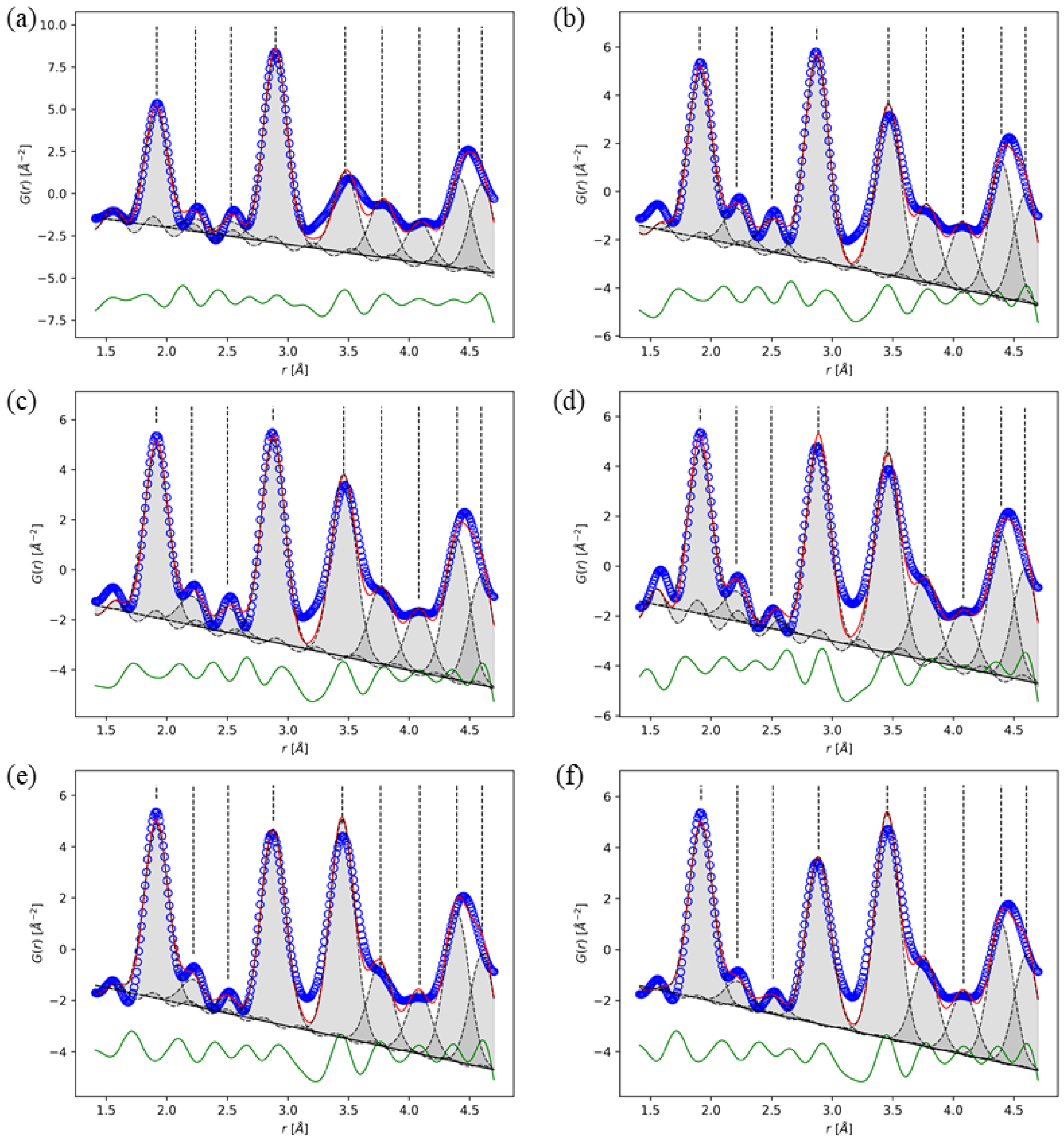

Figure S4. PDF data for parent K_xMnO_2 (a), and δ-MnO_2 floccs heated to 60 (b), 100 (c), 200 (d), 300 (e), and 400 (f) °C.

Table S1. Occupancy parameters extracted from the PDF data by model-free fitting.

	[Mn]	±	[Mn^{IL}]	±	Sum	±
Parent	1.0	-	0.0	-	1.0	-
60 °C	0.74	0.04	0.19	0.04	0.93	0.07
100 °C	0.72	0.04	0.21	0.04	0.93	0.07
200 °C	0.71	0.04	0.28	0.04	0.99	0.08
300 °C	0.67	0.04	0.32	0.04	0.99	0.07
400 °C	0.58	0.04	0.35	0.05	0.93	0.07

CV DATA

Cyclic voltammograms were collected at scan rates of 2, 5, 10, 20, 50, and 100 $mV \cdot s^{-1}$ for δ-MnO_2 floccule electrodes (details of preparation in main text) in aqueous 1 M Na_2SO_4. Error bars on the measured current are the standard deviation of the 4 sample electrodes.

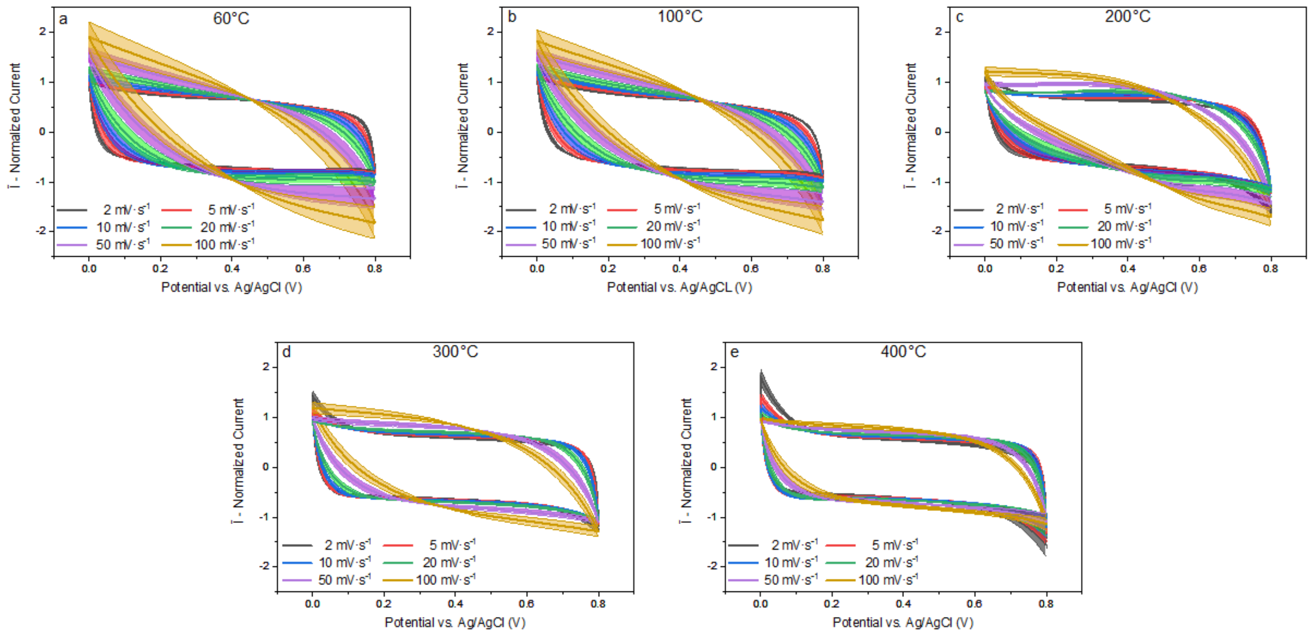

Figure S5. Cyclic voltammograms analyzed in the main text (a-e).

LOGARITHMIC REGRESSION OF B-VALUE

In order to assess the relative pseudocapacitive and capacitive characteristics, the so-called b-values were extracted by logarithmic regression, where the capacitive current is assumed to follow a power-law behavior

$$i = a\, v^{b}. \tag{S1}$$

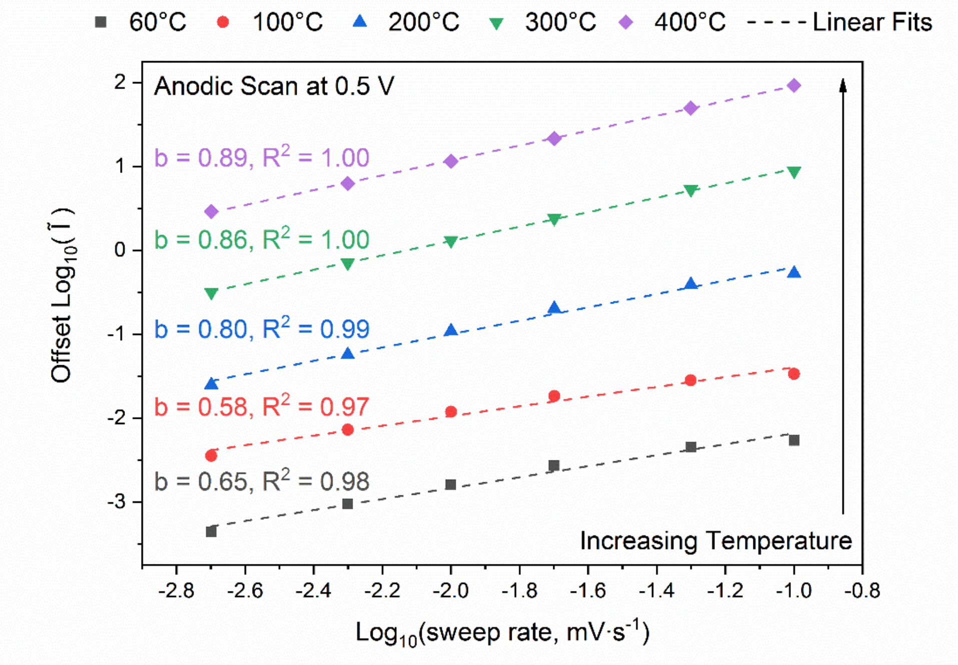

Figure S6. Linear regression determination of the b-value for the anodic scan at 0.5V. Each regression represents a single electrode heat treated at the corresponding temperature.
